# Plague Outbreak in Libya, 2009, Unrelated to Plague in Algeria

**DOI:** 10.3201/eid1902.121031

**Published:** 2013-02

**Authors:** Nicolas Cabanel, Alexandre Leclercq, Viviane Chenal-Francisque, Badereddin Annajar, Minoarisoa Rajerison, Souad Bekkhoucha, Eric Bertherat, Elisabeth Carniel

**Affiliations:** Author affiliations: Institut Pasteur, Paris, France (N. Cabanel, A. Leclercq, V. Chenal-Francisque, E. Carniel);; National Center for Disease Control, Tripoli, Libya (B. Annajar);; Institut Pasteur, Antananarivo, Madagascar (M. Rajerison); University Hospital, Oran, Algeria (S. Bekkhoucha);; World Health Organization, Geneva, Switzerland (E. Bertherat)

**Keywords:** plague, outbreak, reemergence, Yersinia pestis, bacteria, Tobruk, Libya, Oran, Algeria

## Abstract

After 25 years of no cases of plague, this disease recurred near Tobruk, Libya, in 2009. An epidemiologic investigation identified 5 confirmed cases. We determined ribotypes, *Not*1 restriction profiles, and IS*100* and IS*1541* hybridization patterns of strains isolated during this outbreak. We also analyzed strains isolated during the 2003 plague epidemic in Algeria to determine whether there were epidemiologic links between the 2 events. Our results demonstrate unambiguously that neighboring but independent plague foci coexist in Algeria and Libya. They also indicate that these outbreaks were most likely caused by reactivation of organisms in local or regional foci believed to be dormant (Libya) or extinct (Algeria) for decades, rather than by recent importation of *Yersinia pestis* from distant foci. Environmental factors favorable for plague reemergence might exist in this area and lead to reactivation of organisms in other ancient foci.

Plague is a zoonosis caused by the bacillus *Yersinia pestis*. Rodents are the reservoir and fleas are the vector of this organism. Humans most often become infected by an infectious fleabite, which leads to development of a bubonic form of plague (*1*). If the bacillus reaches the lungs, the patient will expel the bacteria while coughing, causing another clinical form: pneumonic plague, which is directly transmissible from person to person. Without prompt and efficient treatment, the case-fatality rate is 40%–70% for bubonic plague and ≈100% for pneumonic plague (*1*).

The plague bacillus is believed to have originated <20,000 years ago in central Asia (*2*), from which it has spread on multiple occasions and caused 3 well-documented pandemics (*1*). The first pandemic, known as Justinian’s plague, reached Africa and then Europe during the sixth century. The second pandemic struck the countries surrounding the Mediterranean in 1348 and then spread rapidly throughout Europe. The third pandemic started in Hong Kong in 1894 and reached previously unscathed territories worldwide. These 3 pandemics were extremely devastating and killed millions of persons. Because of identification of the causative agent (*3*), the reservoir, and the vector of the disease at the end of the 19th century (*4*) and then the availability of effective antimicrobial drugs, human illness and death caused by plague have been considerably reduced since the middle of the 20th century.

However, the disease has not been eradicated. Plague-endemic foci persist in Africa (the most affected continent), Asia, South America, and North America (*5*). Moreover, since the beginning of the 1990s, plague outbreaks have recurred in countries where no cases were reported for decades, and where the disease was believed to have been eliminated. Among the most conspicuous examples is that of India, which experienced a large outbreak of pneumonic plague in 1994 after 30 years with no reports of plague cases (*6*). Another striking example of plague reemergence is that of Algeria, where the most recent cases were reported in the 1940s. After an absence of >50 years, the disease reappeared in 2003 in a village south of Oran (*7**,**8*); and then in 2008, in the Laghouat area (*9*) (Figure 1). Whether these outbreaks were caused by reimportation of the disease from other countries or by reactivation of organisms in a local quiescent plague focus could not be formally established.

**Figure 1:**
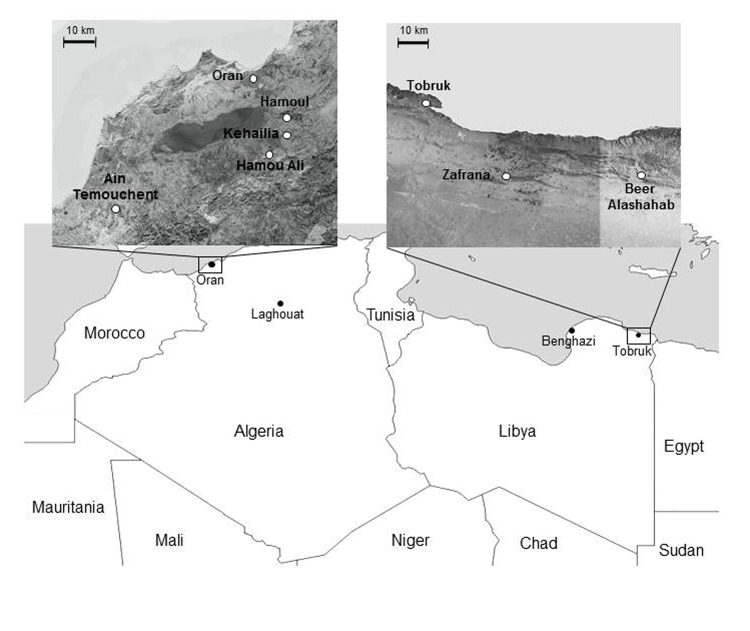
Locations of plague outbreaks in Oran, Algeria, and Tobruk, Libya. Upper panels show regions around Oran and Tobruk where plague cases were found.

Neighboring Libya experienced several plague outbreaks during 1913–1920, the largest of which resulted in 1,449 deaths in Benghazi in 1917 (*10*). Other plague epidemics of lower amplitude occurred in 1972, 1976, 1977, and 1984. After an apparent absence of 25 years, plague cases recurred in June 2009 near Tobruk, Libya, close to the border with Egypt (Figure 1). A total of 5 plague cases were confirmed, 3 of which occurred in 1 family and the other 2 in patients living in the same district (*11**,**12*). An even more recent plague epidemic that had >21 suspected cases was reported in May 2011 in the city of Tobruk (*12*). An investigation of this epidemic did not confirm the plague etiology, but the political troubles resulting from the onset of the revolution in February made this investigation difficult.

The purpose of this study was to obtain insights into the origin of the outbreak that occurred near Tobruk in 2009. We analyzed *Y. pestis* strains that were isolated from patients during this epidemic and determined whether links exist to the recent resurgence of plague in Algeria.

## Materials and Methods

### Bacterial Strains Isolation and Characterization

The *Y. pestis* strains used in this study are shown in the Table. *Y. pestis* strains were isolated from biological specimens after streaking them on cefsulodin-irgasan-novobiocin plates (Merck, Schaffhausen, Switzerland) and injection of bacteria into mice. Phenotypic characterization of strains was determined by biochemical reactions on API 20E and API 50CH strips (bioMérieux, Marcy l’Etoile, France) incubated at 28°C, lysis by a *Y. pestis*–specific bacteriophage, reduction of nitrates, and glycerol fermentation. For further analyses, bacteria were grown in Luria-Bertani broth or on Luria-Bertani agar plates containing 0.002% hemin for 24–48 h at 28°C.

**Table:** Fifteen strains of *Yersinia pestis* used for analysis of plague outbreaks in Libya, 2009, and Algeria, 2003

Strain	Biovar	Country	Place	Year of isolation	Source
IP1973	Medievalis	Libya	Eltarsha	2009	Libyan health authorities
IP1974	Medievalis	Libya	Alashahab	2009	Libyan health authorities
IP1975	Medievalis	Libya	Zafrana	2009	Libyan health authorities
IP516	Medievalis	Iran	Kurdistan	1948	Yersinia Research Unit (Institut Pasteur)
IP519	Medievalis	Iran	Kurdistan	1951	Yersinia Research Unit (Institut Pasteur)
IP562	Medievalis	Iran	Kurdistan	1947	Yersinia Research Unit (Institut Pasteur)
IP564	Medievalis	Iran	Kurdistan	1948	Yersinia Research Unit (Institut Pasteur)
IP565	Medievalis	Turkey	Unknown	1952	Yersinia Research Unit (Institut Pasteur)
IP1860	Orientalis	Algeria	Kehailia	2003	University Hospital, Oran
IP1861	Orientalis	Algeria	Hama Ali	2003	University Hospital, Oran
IP1862	Orientalis	Algeria	Hamoul	2003	University Hospital, Oran
IP1863	Orientalis	Algeria	Ain Temouchent	2003	University Hospital, Oran
IP1864	Orientalis	Algeria	Ain Temouchent	2003	University Hospital, Oran
IP1866	Orientalis	Algeria	Unknown	1944	Yersinia Research Unit (Institut Pasteur)
IP1867	Orientalis	Algeria	Unknown	1945	Yersinia Research Unit (Institut Pasteur)

### Molecular Typing

Total genomic DNA was extracted by using a Gentra Puregene Cell Kit (QIAGEN, Hilden, Germany) according to the manufacturer’s instructions. Ribotyping was performed as described (*13*) after digestion of genomic DNA with *Eco*RI or *Eco*RV restriction enzymes for 30 min at 37°C. Each profile was classified according to the scheme of Guiyoule et al. (*13*).

Insertion sequence–restriction fragment length polymorphism (IS-RFLP) analysis was performed as described (*14*). DNA samples were digested with *Eco*RI (IS*100*-RFLP) or *Hin*dIII (IS*1541*-RFLP) for 30 min at 37°C before being loaded onto 0.8% agarose gels and subjected to electrophoresis for 24 h. The IS fingerprints were analyzed by using BioNumerics software version 6.6 (Applied Maths, Kortrijk, Belgium) as described (*14*). Two IS profiles were considered identical when their percent similarity was >98%.

For pulsed-field gel electrophoresis (PFGE), bacterial genomic DNA was prepared in agarose plugs as described (*15*) and digested with 50 U of *Not*I endonuclease in 200 µL of the corresponding buffer for 3 h at 37°C. Electrophoresis was performed as described (*16*), except that the pulse time ranged from 4 s to 8 s over 42 h in a buffer maintained at 14°C.

## Results

### Plague Outbreak in Libya

On June 9, 2009, three patients 14, 13, and 4 years of age were admitted to Tobruk Central Hospital because of a severe infectious syndrome. All 3 persons were members of the same family of nomads leaving in Eltarsha, 30 km south of Tobruk. The 13-year-old patient (patient 1) had a septicemic syndrome 2 days after admission and died on June 11 despite intensive care. His 14-year-old brother (patient 2) had a tender left cervical lymph node and a fever of 39.5°C. He received ciprofloxacin and doxycycline before being transferred to Benghazi Hospital on June 11, and he recovered. His 4-year-old sister (patient 3) had signs of severe infection with no visible bubo. She received cefotaxime and metronidazole and then gentamicin before being transferred to Benghazi Hospital, and she recovered. Their father reported having axillary lymphadenitis and indicated that 2 or 3 sudden deaths had occurred in the previous 2 months in the region.

On the basis of clinical manifestations and previous plague cases in the Tobruk area, these 3 patients were considered to have contracted plague. All patients from this area with an infectious syndrome were reported as having suspected cases of plague. All but 1 of these patients received gentamicin at Tobruk Hospital and no additional deaths occurred. On June 13, the Libyan authorities reported 13 cases of plague to the World Health Organization (WHO) and requested technical assistance.

A joint investigation of the outbreak led by WHO and the Libyan National Center for Infectious Disease Prevention and Control concluded that the number of plague cases was overestimated, but it identified 2 additional probable cases. These cases were in a 20-year-old woman (patient 4) and a 24-year-old woman (patient 5) who had an infectious syndrome and a painful inguinal lymph node and were admitted to the Tobruk Hospital on June 16 and 18, respectively. Patient 4 lived in Beer Alashahab and patient 5 lived in Zafrana (Figure 1), ≈60 and 30 km from Eltarsha, respectively. Control measures (chemoprophylaxis of contact persons, insecticide treatment, and rodent control) were implemented, and no additional cases were reported.

### Bacteriologic Findings

In Benghazi, blood samples collected from patients 3, 4, and 5 contained gram-negative bacteria resembling *Yersinia* spp. These blood samples and serum samples from all 5 patients and a bubo aspirate from patient 4 were sent to the WHO Collaborating Center at the Institut Pasteur in Antananarivo, Madagascar, for further analyses. F1 dipstick test (*17*) results were positive for samples from all 5 patients. A *Y. pestis* strain was isolated from the blood of patients 3 and 5 and from the bubo aspirate of patient 4, thus confirming the etiology of this outbreak. These 3 strains were then sent to the WHO Collaborating Center at the Institut Pasteur in Paris, France, for further characterization. On the basis of glycerol fermentation and nitrate reduction, the 3 strains were assigned to *Y. pestis* biovar Medievalis (*18*).

### Molecular Characteristics of Strains from Libya

**Figure 2:**
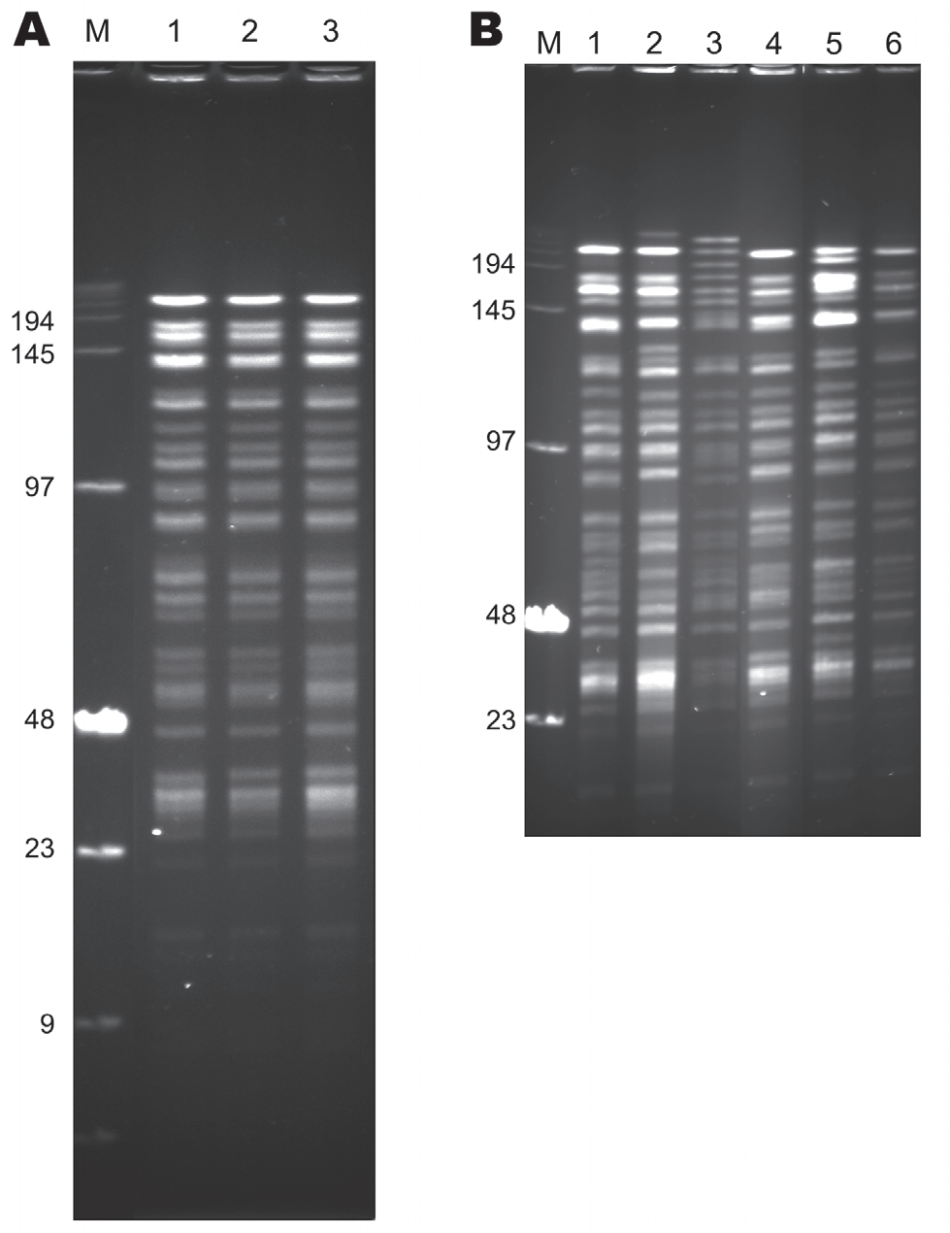
*Not*I pulsed-field gel electrophoresis patterns of *Yersinia pestis* strains of biovar Medievalis obtained during plague outbreak in Libya, 2009. A) Pattern of three 2009 isolates from Libya. Lane M, low-range DNA marker (New England Biolabs, Ipswich, MA, USA); lane 1, IP1973; lane 2, IP1974; lane 3, IP1975. B) Comparison of the pattern of 1 isolate from Libya with those of other biovar Medievalis strains. Lane M, low-range DNA marker (New England Biolabs); lane 1, IP516 (Kurdistan); lane 2, IP519 (Kurdistan); lane 3, IP565 (Turkey); lane 4, IP1975 (Libya); lane 5, IP562 (Kurdistan); lane 6, IP564 (Kurdistan). Values on the left are in kilobases.

**Figure 3:**
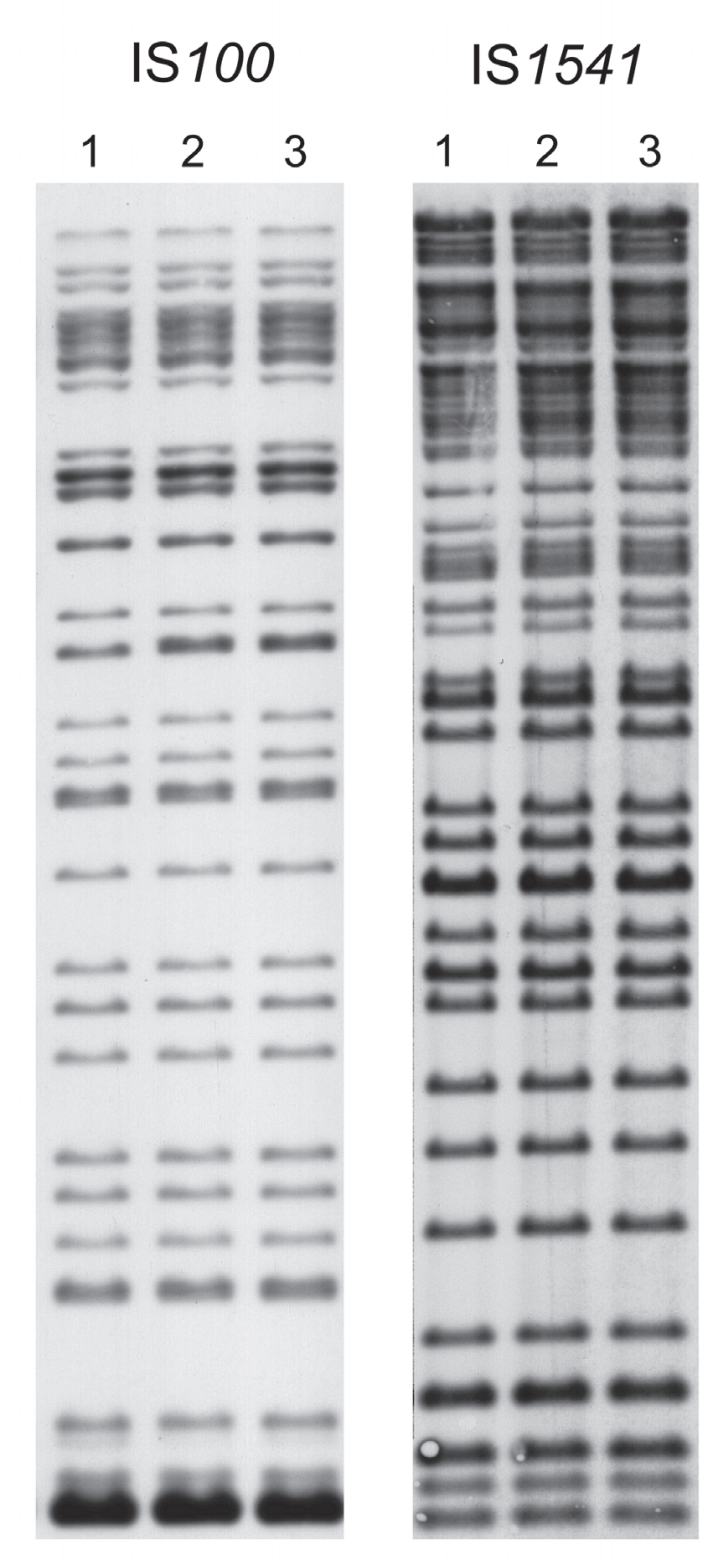
Insertion sequence–restriction fragment length polymorphism profiles of 3 *Yersinia pestis* strains obtained during plague outbreak in Libya, 2009. Genomic DNA of strains IP1973 (lane 1), IP1974 (lane 2), and IP1975 (lane 3) were hybridized with an IS*100* (after *Eco*RI digestion) or an IS*1541* probe (after *Hin*dIII digestion).

The 3 strains exhibited identical *Not*I PFGE profiles (Figure 2, panel A), and all had ribotype O (*Eco*RI.4 + *Eco*RV.5) according to Guiyoule et al. (*13*) and identical IS*100* and IS*1541* hybridization patterns (Figure 3). These results are consistent with a single *Y. pestis* strain as the origin of the cases that occurred in distinct places in the Tobruk area.

**Figure 4:**
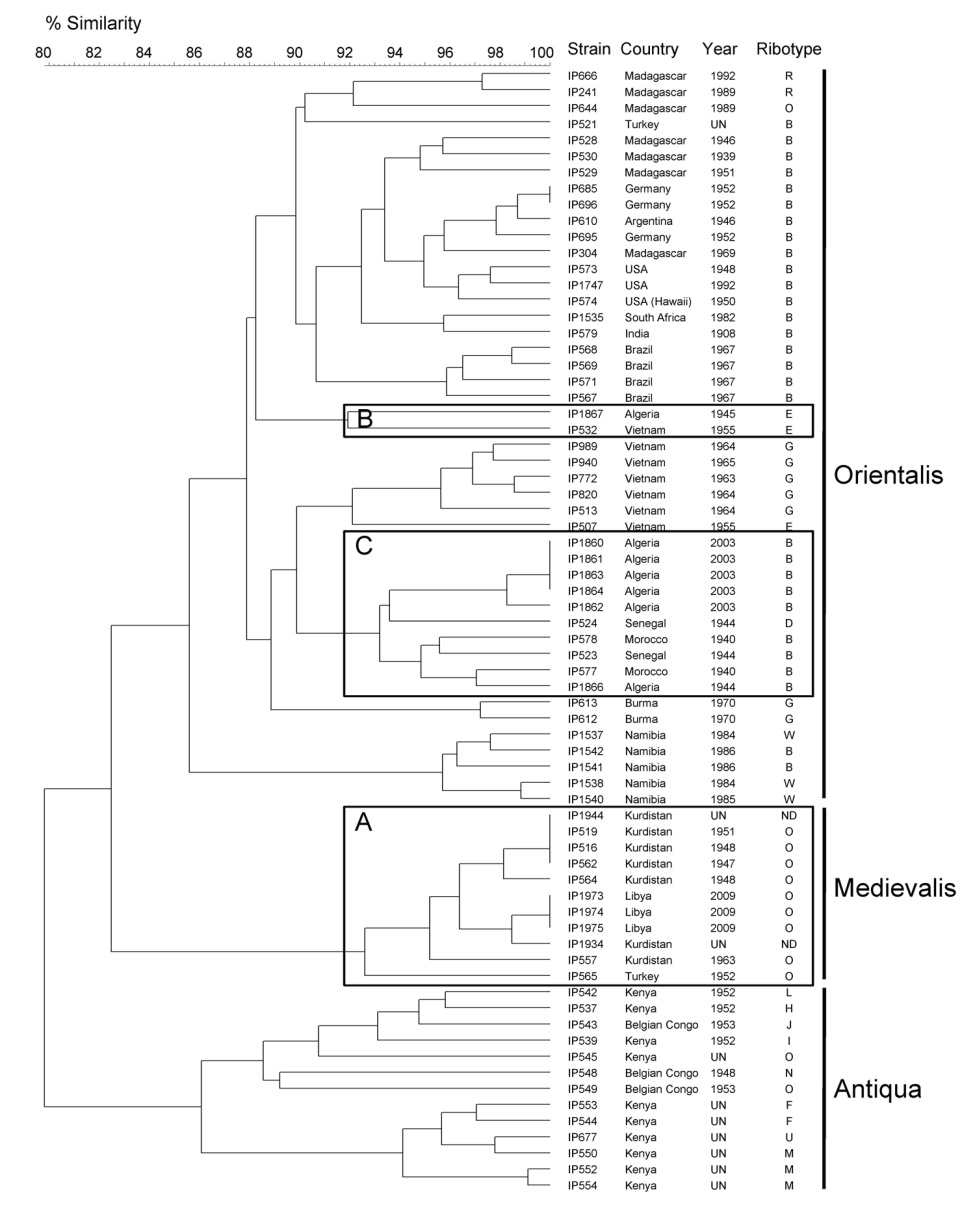
IS*100* and IS*1541* restriction fragment length polymorphism patterns of 70 *Yersinia pestis* isolates of worldwide origin. A) Medievalis branch. B), ancient strain from Algeria (IP1867); C) other strains from Algeria and various isolates from Africa. The dendrogram was constructed by using the unweighted pair group method with arithmetic mean clustering analysis and a position tolerance of 1.8%. Biovar is shown on the right. UN, unknown; ND, not determined.

Ribotype O is commonly associated with biovar Medievalis strains (*13*). When the IS*100* and IS*1541* profiles were combined (2IS-RFLP), the isolates from Libya clustered with other biovar Medievalis strains (Figure 4) in our database (*14*). Moreover, the chromosomal location of some IS*100* sequences determined by PCR was typical of this biovar (*19*). Therefore, our results demonstrate unambiguously that the *Y. pestis* strain that caused the plague outbreak in Libya in 2009 belongs to the biovar Medievalis lineage, a lineage typical for strains that originated in central Asia.

The 2IS-RFLP dendrogram suggested that strains from Libya were closely related to but different from those from Kurdistan and more distantly related to the biovar Medievalis strain from Turkey (Figure 4). A comparison of the *Not*I PFGE profile of an isolate from Libya with those of 5 other biovar Medievalis strains confirmed this observation: all isolates had similar but different profiles (Figure 2, panel B), and the similarity was more pronounced with strains from Kurdistan than with the strain from Turkey. Thus, strains from the 2009 outbreak in Libya are genetically close to those isolated in the Iranian part of Kurdistan.

### *Y. pestis* Strains that Caused the Plague Outbreak in Algeria in 2003

Because *Y. pestis* strains were isolated from patients during the outbreak that occurred in the region of Oran in 2003 (*7*) (Figure 1), we performed the same analyses on these strains and compared them with isolates from Libya. Biochemical characterization of the 5 strains from Algeria indicated that they belonged to biovar Orientalis. They were of ribotype B (*Eco*RI.1 + *Eco*RV.2), which is found only in biovar Orientalis strains (*13*). Their IS*100* + IS*1541* profiles also included them in the biovar Orientalis group (Figure 4). Therefore, the strains that caused the 2003 plague outbreak in Algeria belong to the biovar Orientalis lineage.

**Figure 5:**
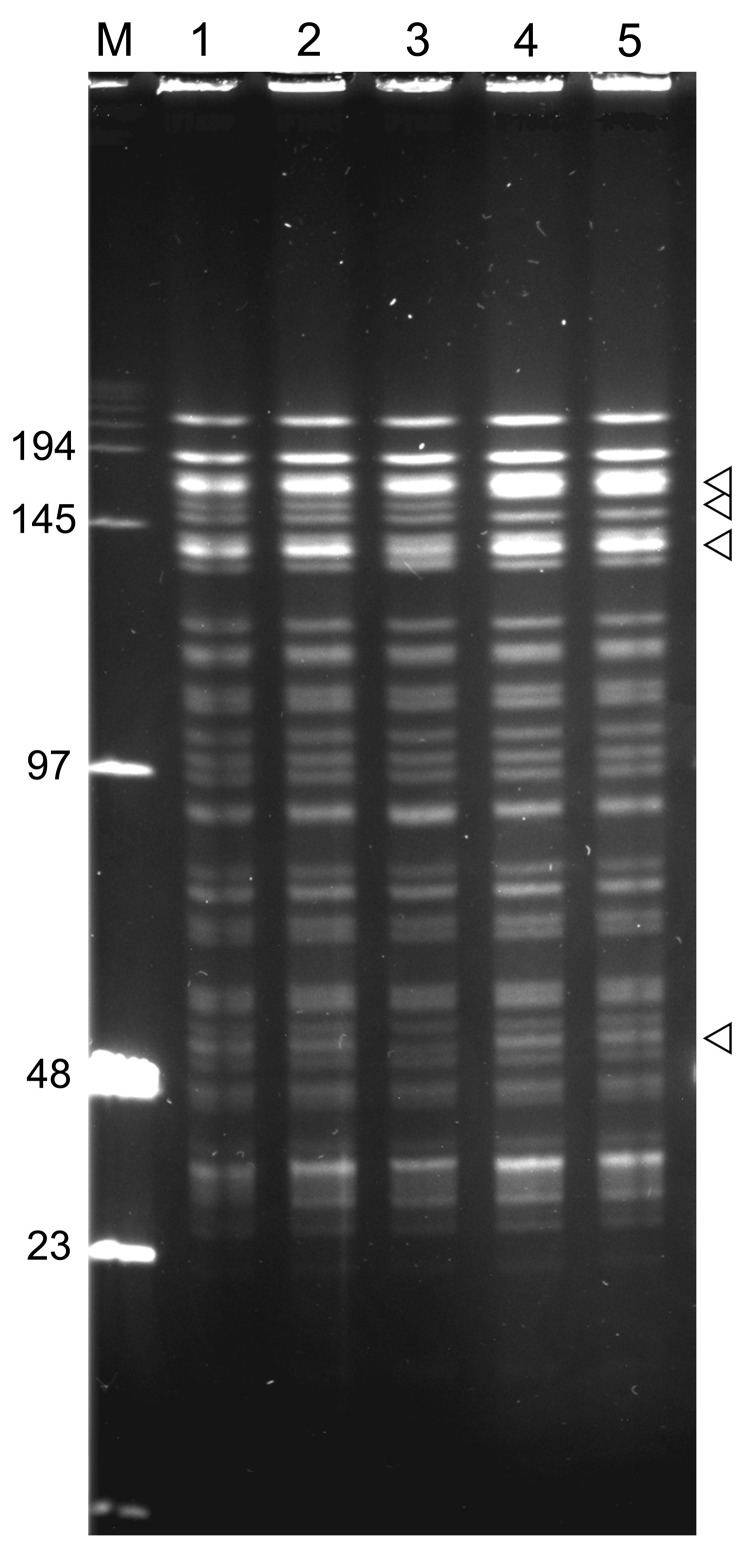
*Not*I pulsed-field gel electrophoresis patterns of *Yersinia pestis* isolates from plague outbreak in Algeria, 2009. Lane M, low-range DNA marker (New England Biolabs, Ipswich, MA, USA); lane 1, IP1860 (Kehailia); lane 2, IP1861 (Hama Ali); lane 3, IP1862 (Hamoul); lane 4, IP1863 (Ain Temouchent); lane 5, IP1864 (Ain Temouchent). Values on the left are in kilobases. Arrowheads indicate positions of variable bands.

Four of these strains had identical IS*100* + IS*1541* profiles, but the IS*1541* profile of the strain from Hamoul (IP1862) displayed 1 additional band. The *Not*I PFGE patterns of the 5 strains from Algeria were highly similar but differed by a few bands (Figure 5). The 2 strains from Ain Temouchent (IP1863 and IP1864) had an identical *Not*I pattern, which was different from that of the strain from Hamoul (IP1862), which also slightly differed from the *Not*I profile of the strains from Kehailia (IP1860) and Hama Ali (IP1861). Thus, these results suggest that the 2003 outbreak in Algeria was caused by closely related, but not identical, strains.

Two *Y. pestis* strains isolated in Oran in 1944 and 1945 from bubonic plague patients were available in our collection and were used for comparison with the 2003 strains. These 2 more ancient isolates also belonged to biovar Orientalis. However, the strain isolated in 1945 (IP1867) had ribotype E, a ribotype found in strains from Saigon, Vietnam. This strain also clustered with an isolate from Saigon (IP532) in the 2IS-RFLP dendrogram (Figure 4), which suggested an epidemiologic link between these 2 foci. In contrast, the strain isolated in 1944 (IP1866) had ribotype B and was found in the same cluster in the 2IS-RFLP dendrogram as that containing the 2003 strains from Algeria (Figure 4). This cluster grouped all other strains from northern Africa (Morocco and Senegal). These data support a local or regional origin of the strains that caused the 2003 plague outbreak in Algeria.

## Discussion

After decades of no plague cases, human plague cases have recently recurred in countries surrounding the Mediterranean Sea. New cases occurred in Saudi Arabia in 1994 (*20*) where no human cases of plague had been reported for ≥40 years, in Jordan in 1997 (*21*), where no cases had been reported during the past 70 years, in Algeria in 2003, where no cases had been reported for 50 years (*7*), and more recently in Libya in 2009, where the last plague case was reported 25 years ago. Recent reappearance of plague cases in this area could have been caused by regional spread of organisms from a single focus, reimportation of strains from distant areas by land or sea transportation, or reactivation of the organism in local foci that had been apparently dormant for years.

Analysis of the 3 strains isolated in Libya in 2009 showed that they were identical by 2IS-RFLP and PFGE, which suggested that a single strain caused this outbreak and that a single focus was the source of infection for different human patients. Phenotypic and genetic analyses indicated that this strain belongs to the biovar Medievalis lineage. Thus, as for other strains of this lineage, the strain from Libya most likely originated in central Asia. To our knowledge, no biovar Medievalis strains have been reported in Africa. However, this finding might reflect a lack of reporting rather than a true absence of these strains in countries in Africa near Asia.

Camel caravans travel through central Asia and the Middle East and consumption of infected camel meat were shown to be responsible for human plague cases in Libya in 1976 (*22*), in Saudi Arabia in 1994 (*20*), in Jordan in 1997 (*21*), and in Afghanistan in 2007 (*23*). Thus, infected camels could have been a means of importing new *Y. pestis* strains into Libya. However, because camels are highly susceptible to plague, it is unlikely that sick animals could travel long distances. Furthermore, dead rats were found in the vicinity of a sick camel in Libya (*22*), *Y. pestis* strains were isolated from rodents and fleas in the corral where a camel died of plague in Saudi Arabia (*20*), and dogs had antibodies against the plague bacillus in Jordan (*21*). These observations suggest that plague was already present in these countries and that camels were not the mode of transport.

Resurgence of plague in Libya in 2009 could most likely be attributed to reactivation of established and permanent local plague foci resulting from ancient importation from central Asia. Strengthening this hypothesis are the numerous plague outbreaks that occurred in Libya during the 20th century. In the Tobruk area, human cases were reported in 1976–1977 and in 1984 (*24*). This finding and the fact that the recent epidemic involved persons living 30–60 km from each other are highly evocative of reactivation of organisms in a local plague focus in 2009. Comparison of strains from past epidemics in Libya with the strain from 2009 would have helped answer this question, but such strains were not available in our collection.

The sudden resurgence of plague cases in the Tobruk region >2 decades after the last reported case might be linked to unusual climatic conditions. The outbreak was preceded by a particularly humid winter, which favored flea proliferation, and exceptionally good harvests, which supported rodent multiplication. The effect of climatic changes on human plague has been documented (*25**,**26*) and further emphasizes the need to take into consideration the effect of global warming on infectious diseases that have a nonhuman reservoir.

Although Libya and Algeria have a common border, our results demonstrate that the plague outbreaks that occurred recently in Algeria were not caused by spread of organisms from the focus in Libya. Phenotypic and genetic analyses of 5 strains isolated from patients in Algeria in 2003 demonstrated that they belong to biovar Orientalis. Similarly, the strains that caused the plague episode in Laghouat (550 km south of Algiers) in 2008 had a multispacer sequence type typical for biovar Orientalis strains (*9*). This lineage is distinct from the biovar Medievalis lineage of strains from Libya. Thus, the plague foci in Algeria and Libya are not linked.

Several plague outbreaks in Oran have been attributed to importation of infected rodents or fleas by marine shipping, e.g., during World War II military operations (*27*). Ribotyping and 2IS-RFLP analyses of the *Y. pestis* strain isolated from a bubonic plague patient in the Oran area in 1945 fully support this point and suggest that this strain was imported from southern Vietnam (Saigon). In contrast, *Y. pestis* isolates from 1944 and from 2003 in Oran cluster together by 2IS-RFLP. They also cluster with the other strains from northern Africa (Morocco and Senegal) isolated in the 1940s. These results are consistent with reactivation of organisms in a local or regional plague focus and argue against importation of infected materials or animals from a distant plague-infected region.

Also arguing for existence of an active local reservoir was detection of *Y. pestis* DNA in fleas collected near Oran 1–2 years after the 2003 outbreak (*8*) and in native rodents trapped in the Laghouat area a few months after the 2008 plague cases (*9*). The fact that 3 similar but distinct *Not*I patterns were observed among the 5 *Y. pestis* strains isolated in Oran in 2003 also argues against importation of a foreign strain and suggests emergence of variants from a local common ancestor. If true, this suggestion would also imply that it was not organisms in 1 focus but organisms in several adjacent foci that were reactivated at the same time in the Oran region. Climatic and environmental factors may have played a critical role in this resurgence because they have been shown to be predictors of human risk for exposure to plague in other foci in Africa (*28*).

Our results indicate that adjacent but independent plague foci coexist in Algeria and Libya. Plague outbreaks that occurred in these 2 countries are most likely the result of reactivation of organisms in local foci that were believed to be dormant (Libya) or extinct (Algeria). Recent reemergence of these independent foci suggests that climatic and environmental changes in northern Africa may be favorable for the *Y. pestis* epidemiologic cycle. Thus, other countries in northern Africa that have had plague foci may also be at risk for plague outbreaks in the near future.
